# Integrative Management of Cancer Pain: A Scoping Review of the Literature

**DOI:** 10.1002/cam4.70833

**Published:** 2025-05-03

**Authors:** Brieze K. Bell, Jaeyoon Cha, Kathleen A. Cavanaugh, David L. O'Riordan, Michael W. Rabow, Adrienne K. Yang, Sohil Patel, Sa Heen Park, Megan K. McGrath, Evans M. Whitaker, Sarah S. Nouri, Stephanie W. Cheng

**Affiliations:** ^1^ University of California San Francisco San Francisco California USA; ^2^ Harvard Medical School Boston Massachusetts USA; ^3^ The MERI Center for Education in Palliative Care, Supported by the Mount Zion Health Fund San Francisco California USA; ^4^ Stanford University School of Medicine Palo Alto California USA

**Keywords:** acupuncture, cancer pain, complementary and alternative medicine, integrative health, integrative medicine, integrative oncology, mindfulness

## Abstract

**Background:**

Cancer‐related pain is common and debilitating. Patients frequently use integrative medicine therapies to manage this, though safety and efficacy evidence is incomplete. This scoping review aims to characterize the state of integrative cancer pain therapy (ICPT) and identify priorities for future research.

**Methods:**

Following PRISMA guidelines, we searched PubMed, Embase, Web of Science, PsycINFO, CINAHL, and Cochrane for ICPT studies published between January 1, 1975 and May 26, 2022. Study findings were extracted and analyzed using descriptive statistics and thematic analysis. Interventions were categorized as follows: Whole Systems of Medicine (WSM); Mind–body Medicine (MBM); Botanicals and Supplements (BAS); and Manual Therapies (MT). Quality appraisal was performed using the Downs and Black checklist. Efficacy was “positive” if there were statistically significant differences between study arms (*p* < 0.05) favoring ICPT.

**Results:**

Among 1246 studies reviewed, 151 met inclusion criteria; 63.5% were excellent or good quality, and 68.9% were RCTs; 122 studies (80.7%) were published since 2010. Studies occurred in 24 countries, in variable settings, among participants with a wide range of cancers, disease status, and age ranges. Studies investigating WSM and MBM interventions were most frequent (35.7% for each), MT (20.59%), and BAS (7.9%). Overall, of the included studies, 127 (84.1%) found that the ICPT intervention reduced pain.

**Conclusions:**

Studies on ICPT are increasingly common, and the majority of ICPT interventions demonstrated a positive impact on cancer pain. Future rigorous research should compare efficacy across integrative and biomedical interventions and explore how to incorporate evidence‐based ICPT into standard cancer treatment.

## 1 Introduction

Pain is a common challenge for persons living with cancer, affecting up to 66% of those with active cancer and nearly 40% of cancer survivors [1, 2]. Poorly controlled cancer pain negatively impacts quality of life [3] and can create a barrier to cancer treatment adherence [4]. Opioids and other pharmacologic therapies play an important role in the treatment of cancer pain, but their use comes with substantial side effects and risks [5, 6, 7].

Increasingly, people living with cancer in the United States and around the world are incorporating integrative therapies into their cancer symptom management regimens, including to help control pain [8]. Integrative Medicine is a holistic approach to healing that emphasizes whole‐person care, combining the best of biomedicine with evidence‐informed, complementary therapies to help individuals optimize their health and well‐being [9, 10, 11]. Several healing modalities fall under the Integrative Medicine umbrella. The National Center for Complementary and Integrative Health (NCCIH) created a five‐domain paradigm to categorize integrative therapies, which includes (1) manipulative and body‐based methods, (2) mind–body medicine, (3) alternative medical systems, (4) energy therapies, and (5) biologically based therapies [10, 12]. This paper uses a framework modeled on this five‐domain paradigm.

Recognizing the added value of integrative modalities in the care of people with cancer, “Integrative Oncology” has emerged as a subspecialty available at many U.S. comprehensive cancer centers that seek to incorporate acupuncture, mindfulness, nutrition counseling, exercise, and other evidence‐informed lifestyle therapies into conventional cancer treatment [13]. Recent surveys indicate that up to 40% of cancer patients and cancer survivors used some form of integrative medicine in the previous year [8, 14]. Nonetheless, many patients do not disclose integrative medicine use to their medical teams [15], and providers cite inadequate knowledge as a barrier to incorporating these therapies into clinical practice [16, 17].

There is a need for clarity regarding the evidence for efficacy of integrative cancer pain therapies (ICPT). The Society for Integrative Oncology (SIO) and American Society of Clinical Oncology (ASCO) recently published guidelines for integrative management of cancer pain based on randomized controlled trials (RCTs), systematic reviews, and meta‐analyses published between 1990 and 2021 [18]. A small number of prior systematic reviews have evaluated the efficacy for complementary therapies to treat cancer‐related pain [19, 20]. These important contributions to the literature provide valuable clinical guidelines as well as a summary of some of the evidence for these therapies in treating cancer pain. This scoping review offers a vital complement to prior published reviews and to the SIO‐ASCO guidelines by providing a comprehensive overview of the ICPT literature, describing study characteristics, intervention modalities, statistical outcomes in this field, and opportunities for future research. This review includes more studies published in an expanded timeframe of 1975–2022 and incorporates non‐randomized pragmatic studies in addition to RCTs, which provides a more complete picture of the current field of ICPT. Including these additional studies enhances our ability to understand the real‐world application of integrative medicine therapies for cancer pain, expands our understanding of the potential evidence base for these therapies, and provides more clarity regarding directions for future research.

## 2 Methods

### 2.1 Study Design

We used the 2020 Preferred Reporting Items for Systematic reviews and Meta‐Analyses (PRISMA) scoping review extension (PRISMA‐ScR checklist can be found in Appendix S1) [21]. The goal of this scoping review is to provide a preliminary assessment of the scope of available research on integrative cancer pain therapies. Given this objective, this review includes a broad range of study types including randomized controlled trials, cohort studies, pre/post‐test studies, and case series. The study differs from a systematic review as its primary goal is to identify key concepts, themes, and gaps in this area of the literature to inform a current understanding of the field and pave the way for future rigorous studies on this topic.

### 2.2 Search Strategies

With a health sciences librarian, we developed a search strategy across MEDLINE/PubMed, EMBASE, Web of Science, PsycINFO, CINAHL, Cochrane, and manual searches (Table S1).

### 2.3 Selection of Studies

#### 2.3.1 Database Search

We restricted publications to clinical studies of humans published between January 1, 1975, and May 26, 2022, that met the following inclusion criteria: English language, adult populations, utilized an integrative therapy (e.g., mind–body, movement therapy, manual medicine, spirituality, energy medicine, dietary intervention, botanicals/supplements, or whole systems approach), included an outcome related to cancer‐related pain (e.g., pain score, measurement, functional status), included primary data, had a participant *N* ≥ 5, and published in a peer‐reviewed journal. We defined cancer‐related pain as pain related to cancer burden or cancer treatments (as opposed to chronic nonmalignant pain in patients with cancer). We excluded publications that did not meet the above inclusion criteria, as well as conference abstracts, reports, and other “gray” literature. One author (E.W.) reviewed titles and removed duplicates. To ensure interrater reliability, three authors (B.B., K.C., and S.C.) reviewed and selected abstracts for full data extraction using RAYYAN software. Discrepancies were resolved by consensus.

#### 2.3.2 Manual Search

Articles that met inclusion criteria were imported into Web of Science, and all “cited” and “cited by” publications were reviewed. In addition, for articles that came up during the database search that did not meet inclusion criteria but were thought to potentially include references that may meet inclusion criteria (e.g., review articles), Web of Science was again utilized to review “cited” and “cited by” publications. Publications in the above two categories that seemed to meet inclusion criteria were reviewed by the same three authors as above in RAYYAN, and abstracts that were confirmed to meet all inclusion criteria were selected for full data extraction. Discrepancies were again resolved by consensus.

### 2.4 Data Extraction and Analysis

We created a data extraction tool to chart the following: article characteristics (authors, journal, and year published), population and setting, study type, intervention(s), pain assessment tool(s)/scale(s) used, outcomes, and relative statistical findings. Interventions were categorized into four modalities (based on the non‐biologically based modalities of the NCCIH model) [12]: Whole Systems of Medicine (WSM), including acupuncture; mind–body medicine (MBM); botanicals and supplements (BAS), including cannabis; and manual therapies (MT), which were further divided into 19 submodalities (Appendix S2). Efficacy was defined as “positive” if there were statistically significant differences between study arms (*p* < 0.05). For each publication, two reviewers performed data extraction and assessed methodological quality using the previously validated Downs and Black checklist [22]. This checklist is a validated, reliable, 27‐item quality assessment tool that is designed to provide an overall quality score for both randomized and non‐randomized studies, making it an ideal quality assessment tool for a scoping review. It includes five categories of quality assessment, including questions pertaining to study quality, external validity, study bias, confounding and selection bias, and study power. Discrepancies were resolved by consensus. Downs and Black score ranges were categorized into quality quartiles (excellent, good, fair, poor) as previously reported in the literature [23].

## 3 Results

**FIGURE 1:**
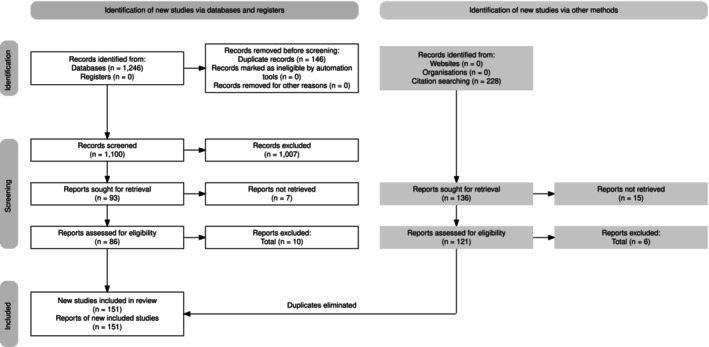
PRISMA flow diagram [24]. Studies were excluded if they were not original research, if the population was not cancer‐specific, did not focus on cancer pain as a primary outcome, did not provide an integrative intervention for pain, were not conducted on human subjects, were written in languages other than English, or contained fewer than five participants.

A comprehensive search of six databases initially identified 1246 studies. Studies that did not meet inclusion criteria were excluded. Once duplicates (146) were eliminated and studies were screened according to the above criteria, 151 studies met inclusion criteria (Figure 1). A complete list of included studies can be found in Table S2.

### 3.1 Study Characteristics

**FIGURE 2:**
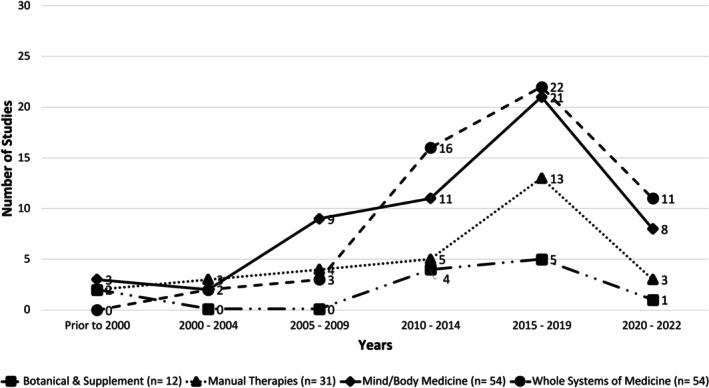
Frequency of studies published by modality.

Studies included in the analysis were conducted in 24 countries, with 56 (37.1%) occurring in the United States and 14 (9.3%) occurring in China. Studies also took place in several other countries throughout Asia, Europe, North America, and Australia, with four studies (2.6%) occurring in multiple countries and five studies in which the geographic setting was not specified (3.3%) (Table S3). Studies occurred in outpatient (39.7%), inpatient (33.8%), and home hospice (8.6%) settings, with 17.2% of studies taking place in mixed or unspecified settings. Studies included a wide range of participants and cancer types, with 54.3% of participants receiving active cancer therapy during the study period, 6.6% in survivorship, 3.3% in palliative care/hospice, and 35.8% with mixed or unspecified treatment status. Study size and design were heterogeneous, with 45% of studies containing fewer than 50 participants, 31.8% containing 50–99 participants, and 23.2% containing 100 or more. Of the included studies, 68.9% were RCTs, 17.2% were cohort studies, 12.6% were pre/post‐test studies, and 1.3% were case series; 76.2% of studies had a comparison group. The vast majority (93.4%) of studies reported the biological sex of study participants, while a smaller subset reported additional demographic details (Table 1). The frequency of study publication in all four modalities increased substantially after 2005, with 121 studies (80.1%) having been published since 2010 (Figure 2). Notably, study publication frequency declined between 2020 and 2022 in all four modalities.

**TABLE 1:** Study characteristics.

Characteristics	Frequency
	*N* (%)
	*N* = 151
Modality	
Whole systems of medicine	35.7 (54)
Mind body medicine	35.7 (54)
Manual therapies	20.5 (31)
Botanicals	7.9 (12)
Cancer status of patient population	
Active treatment	54.3 (82)
Survivorship	6.6 (10)
Palliative	3.3 (5)
Mixed	17.9 (27)
Not specified	17.9 (27)
Sample size:	
< 50	45.0 (68)
50–99	31.8 (48)
100+	23.2 (35)
Comparison group present (Yes)	76.2 (115)
Study setting	
Outpatient	39.7 (61)
Inpatient	33.8 (51)
Home/hospice	8.6 (13)
Mixed	7.9 (12)
Not specified	9.3 (14)
Patient characteristics reported	
Sex	93.4 (141)
Race	39.7 (60)
Study type	
RCT	68.9 (104)
Cohort	17.2 (26)
Pre/post‐test	12.6 (19)
Case series	1.3 (2)
Study efficacy	
Positive	84.1 (127)
Negative	15.2 (23)
Mixed results^a^	0.7 (1)

^a^ Weinrich, S: This article found a significant decrease in pain for male subjects but no change for female subjects.

### 3.2 Intervention Modality

Among the four broad categories of integrative interventions, including whole systems of medicine (WSM), mind–body medicine (MBM), manual therapies (MT), and botanicals and supplements (BAS), the largest categories were WSM and MBM, which accounted for 54 studies (35.7% for each) and included acupuncture, acupressure, electroacupuncture, reflexology, and moxibustion for WSM, and a broad range of interventions including hypnosis, relaxation, mindful movement practices, art/music therapy, and psychotherapy for MBM. There were 31 MT studies (20.5%), which included massage therapy, electrical stimulation (TENS), Reiki, and therapeutic touch. There were 12 BAS studies (7.9%), which focused primarily on interventions using cannabis (10 studies), and one study each on cholecalciferol supplementation (vitamin D) [25] and sesame oil [26].

The most commonly studied ICPT were acupuncture (*n* = 32), massage therapy (*n* = 16), physical exercise (*n* = 12), art or music therapy (*n* = 12), and cannabis (*n =* 10).

### 3.3 Study Quality and Intervention Efficacy

**FIGURE 3:**
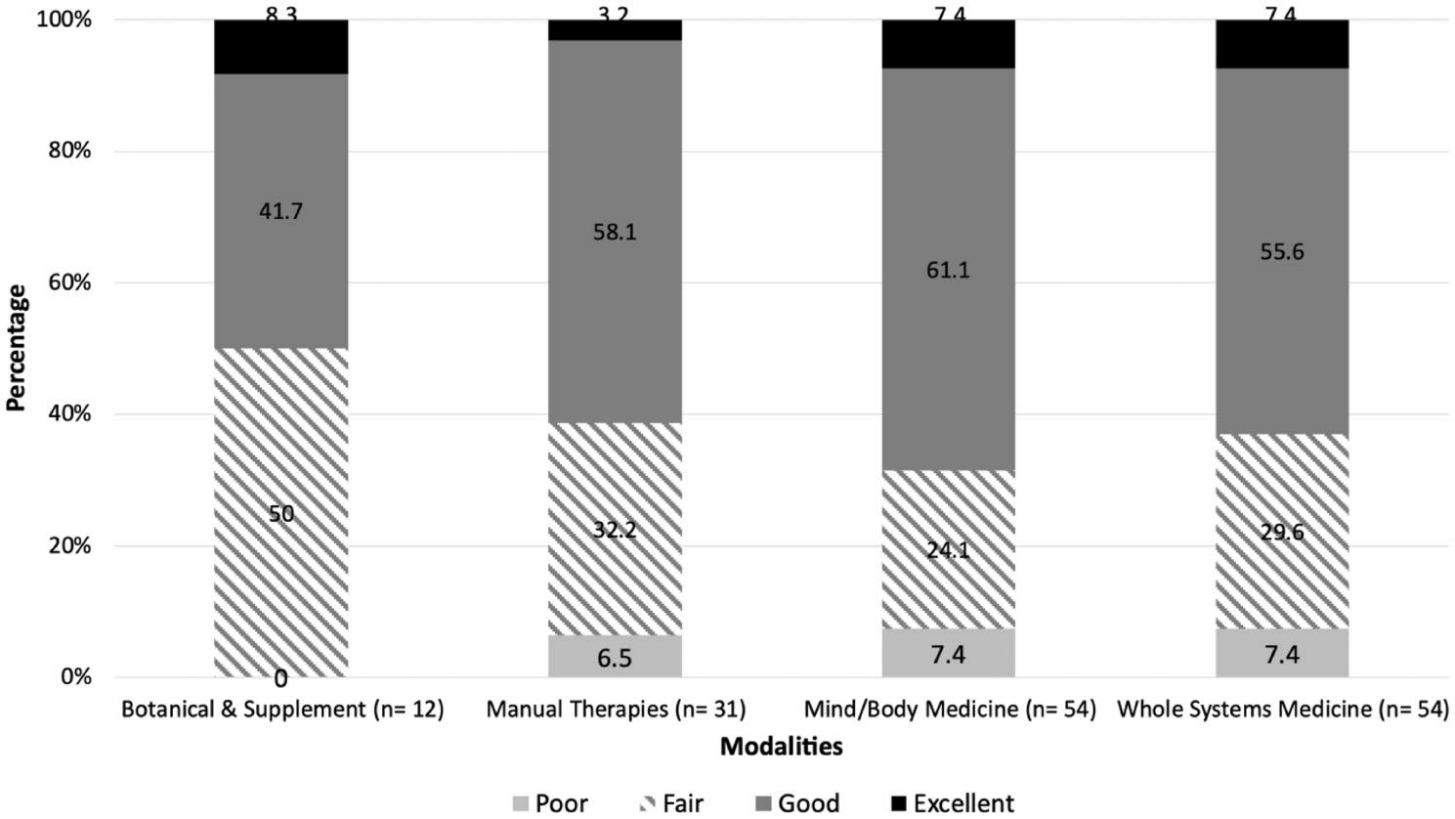
Publication quality by modality.

Overall, 96 (63.5%) of the included studies were rated as excellent or good quality according to the Downs and Black checklist. Specifically, in the WSM category, 34 studies (61.8%) were rated as excellent or good. Within the MBM category, 37 studies (68.5%) were rated as excellent or good. In the MT category, 18 studies (60%) were rated as excellent or good. In the BAS category, six studies (50%) were rated as excellent or good (Figure 3).

**FIGURE 4:**
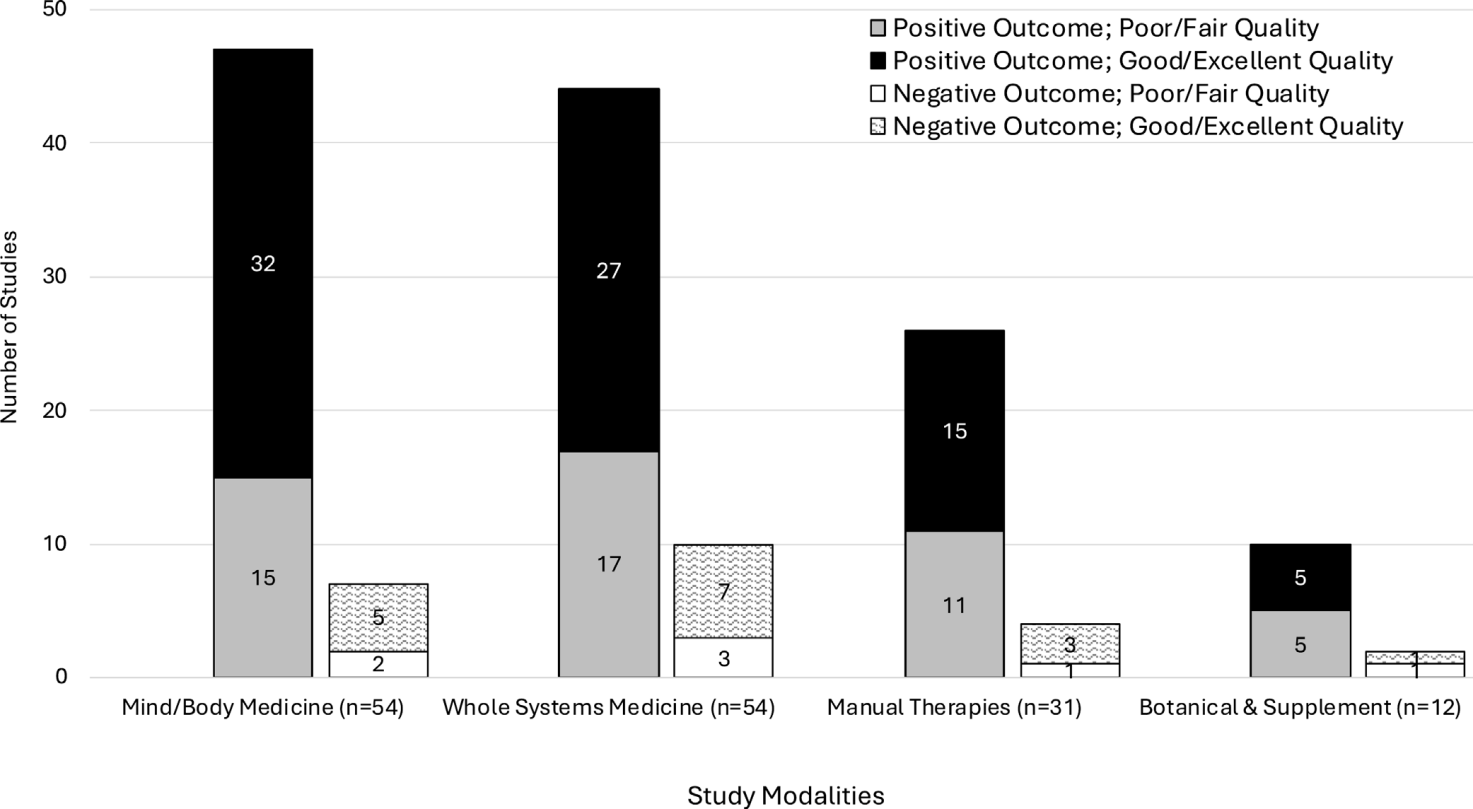
Efficacy on pain by modality and study quality.

Of the included studies in all categories, 127 (84.1%) found that the integrative intervention had a positive impact on cancer pain. By category, 44 WSM studies (81.5%), 47 MBM studies (87%), 26 MT studies (83.9%), and 10 BAS studies (83.3%) demonstrated reductions in pain (Figure 4). Among studies of good or excellent quality only, findings were similar: 79 (83.2%) studies overall (79.4% WSM studies, 86.5% MBM studies, 83.3% MT studies, and 83.3% BAS studies) found that the integrative intervention had a positive impact on cancer pain.

Our analysis revealed the utilization of 46 distinct pain assessment tools across the 151 included studies (Table 2). Most used a single instrument to assess pain (*n* = 109, 72.1%) and nearly all used well‐recognized, validated scales: the Numeric Rating Scale (NRS) (*n* = 50, 32.9%), the Visual Analog Scale (VAS) (*n* = 42, 27.6%), the Brief Pain Index (BPI) (*n* = 19, 19.1%), and the Edmonton Symptom Assessment Scale (ESAS) (*n* = 14, 9.2%). Only three studies reported their pain assessment instrument as “study specific,” [27, 28, 29] and one study reported the use of an unspecified Likert scale [30].

**TABLE 2:** Pain assessment tools.

Instruments	*N* (%)
Mean number of instruments	1.37
Median	1
Range	1–4
Numeric rating scale (NRS)	50 (32.9)
2 Visual analogue scale (VAS)	42 (27.6)
3 Brief pain index (BPI)	19 (19.1)
4 Edmonton symptom assessment scale (ESAS)	14 (9.2)
5 Magill pain questionnaire	8 (5.2)
6 Qualitative	5 (3.3)
7 Study Specific	3 (2.0)
8 MD Anderson Symptom Inventory (MDASI)	3 (2.0)
9 Shoulder pain & disability index	3 (2.0)
10 Common terminology for adverse events	2 (1.3)
11 Dichotomous response	2 (1.3)
12 Wong–Baker Faces	2 (1.3)
13 Hospital anxiety and depression scale (HADS)	2 (1.3)
14 Prescription/Meds Documentation	2 (1.3)
15 Neuro pain scale	2 (1.3)
16 Present pain intensity	2 (1.3)
17 Diary	2 (1.3)
18 Memorial pain assessment card	2 (1.3)
19 Pain relief reduction score	1 (0.7)
20 Karnofsky performance status (kps)	1 (0.7)
21 Pain reduction and pain relief score	1 (0.7)
22 Likert scale (Unspecified)	1 (0.7)
23 EORTC_QLQ_C30	1 (0.7)
24 Patients' global impression of change (PGIC)	1 (0.7)
25 Pain scale by JCAHO	1 (0.7)
26 Multi‐dimensional pain inventory (MPI)	1 (0.7)
27 Chinese memorial symptom assessment scale (MSAS)	1 (0.7)
28 Visual assessment	1 (0.7)
29 Pain self‐efficacy questionnaire (PSEQ)	1 (0.7)
30 Profile of mood states	1 (0.7)
31 Pain medications preference scale (PMPS)	1 (0.7)
32 Treatment satisfaction questionnaire	1 (1.3)
33 Sheehan disability scale (SDS)	1 (0.7)
34 Functional assessment cancer therapy	1 (0.7)
35 Patient specific functional threshold	1 (0.7)
36 Pressure pain threshold	1 (2.6)
37 Medical outcomes study pain (MOS Pain)	1 (0.7)
38 Douleur Neuropathique en 4 Questions (DN 4)	1 (0.7)
39 Pain thermometer	1 (0.7)
40 Pain detect	1 (0.7)
41 German pain questionnaire	1 (0.7)
42 Breast cancer prevention trial musculoskeletal subscale (BCPT_MS)	1 (0.7)
43 Australian/Canadian osteoarthritis hand index (AUSCAN)	1 (0.7)
44 EuroQol 5 Dimension (EQ5D)	1 (0.7)
45 Pain assessment tool (PAT)	1 (0.7)
46 Skilled nursing visit report (SNVR)	1 (0.7)

## 4 Discussion

Patients with cancer commonly utilize integrative medicine therapies to help with their recovery, symptom management, and quality of life, but our understanding of the scope of the evidence for their use has been limited [8, 31, 32, 33]. This scoping review of ICPT is the first attempt of which we are aware to comprehensively characterize the literature body of ICPT studies published since 1975, incorporating non‐randomized pragmatic trials in addition to RCTs to reflect real‐world applications of these therapies. The trends observed in this review reflect the growing popularity of using integrative medicine modalities for cancer pain by patients and clinicians, as well as efforts to expand scientific study of their efficacy.

The majority (84.1%) of ICPT studies found that the integrative intervention being researched had a positive impact on cancer pain. This trend was particularly pronounced for acupuncture interventions, which is consistent with recently published cancer pain treatment guidelines [18]. Acupuncture and acupressure interventions were generally found to improve several kinds of cancer‐related pain, including general cancer pain [34, 35, 36, 37, 38], postoperative pain [39, 40, 41], and aromatase inhibitor‐related arthralgias [42, 43, 44]. Some studies found that acupuncture reduced the need for pain medication use during cancer treatment [30, 45].

Several studies examining mindfulness interventions also demonstrated a positive impact on cancer‐related pain, including 5/5 hypnosis studies and 9/10 relaxation studies [46, 47, 48, 49, 50, 51, 52, 53, 54, 55, 56, 57, 58]. Nine of 10 studies that used cannabis as an intervention demonstrated improvements in cancer pain [28, 59, 60, 61, 62, 63, 64, 65], though one older study cited sedation as a barrier to therapeutic benefit at higher doses of THC (in excess of 15–20 mg per dose) [28]. The movement‐based therapies included in this analysis were varied, ranging from water therapy to yoga, dance, physical therapy, and walking exercise. Yoga in particular was found to improve cancer pain in multiple studies [66, 67, 68].

Frequency of study publication declined in all four modalities between 2020 and 2022. This may reflect the early impact of the COVID‐19 pandemic on original research efforts in this arena, which is consistent with trends in non‐COVID‐related academic medical research worldwide during this period [24, 69].

As compared to prior published reviews and guidelines on this topic [18, 19, 20], this scoping review expands the type and number of studies examined, the years included and the level of detail on quality and outcomes for integrative therapies in the treatment of cancer pain. It reflects the potential for acupuncture, mindfulness and movement‐based therapies in particular to have a positive impact on cancer pain and illustrates that these modalities are strong candidates for future rigorous research in this field.

### 4.1 Limitations

The goal of this scoping review is to provide a comprehensive overview of the current state of integrative therapies for cancer pain. As such, authors intentionally included a broad temporal and methodological range of studies to identify key themes, gaps, hypotheses, and targets for future rigorous research on this topic. While this approach paints a clear picture of the current state of the field, it limits our ability to draw definitive conclusions on the efficacy of these interventions for cancer pain. Given the language proficiency of our authorship team, we could only include English‐language publications; future evaluation of non‐English‐language publications may provide a more complete assessment of the efficacy of ICPT. Nevertheless, nearly two‐thirds of included studies were conducted outside the United States.

Although 63.5% of studies included in this review were rated as excellent or good quality according to the Downs and Black checklist, this body of literature is highly heterogeneous. Study design, size, cancer type, clinical condition and geographic setting were variable. Many pilot studies aimed to assess the feasibility of the intervention rather than efficacy. A wide range of pain assessment tools was used; some of which measured pain indirectly (e.g., emotional distress related to pain) or may have been impacted by confounders (e.g., change in renal function impacting medication dosing), though these were used in < 10% of studies. We chose to include pragmatic observational studies in addition to RCTs to reflect the real‐world application of integrative medicine therapies, which are often underfunded and difficult to study in a rigorous way. As a result, some of the studies reviewed lack methodological rigor. While some studies included demographic data such as age range, biological sex of study participants, only a minority of studies reported on additional demographic details such as race and ethnicity.

We sorted studies into four large categories, based on a widely utilized format. As there were a limited number of studies on Reiki and Therapeutic Touch (*n* = 2), we analyzed these studies as part of the Manual Therapy modality. We recognize, however, that these interventions are often classified differently, for example, as energy therapy. Several studies failed to report adverse events, and very few acknowledged that cost and time may be significant barriers to widespread treatment implementation.

## 5 Conclusion and Future Directions

Studies on ICPT are increasingly common, but characteristics remain varied. The majority of studies included in this review demonstrated that ICPT interventions reduce cancer pain. Given the promising findings highlighted in this review, the significant public health burden created by cancer pain, and the widespread patient interest in and use of integrative medicine therapies for pain management, there is an imperative to conduct additional rigorous and well‐funded research on these interventions and to incorporate integrative medicine therapies more thoughtfully and consistently in the armamentarium of evidence‐based cancer pain treatment options. Future research should explore comparative efficacy across integrative and non‐integrative interventions and effective implementation of ICPT into standard cancer pain treatment protocols. In continuing to build the research base for ICPT, innovation and access (including insurance coverage) should continue to grow as well.

## Author Contributions

**Brieze K. Bell:** conceptualization (equal), data curation (equal), formal analysis (equal), funding acquisition (supporting), investigation (equal), methodology (equal), project administration (equal), supervision (supporting), visualization (supporting), writing – original draft (lead), writing – review and editing (supporting). **Jaeyoon Cha:** data curation (supporting), formal analysis (supporting), project administration (supporting), visualization (supporting), writing – review and editing (supporting). **Kathleen A. Cavanaugh:** conceptualization (supporting), investigation (supporting), methodology (supporting), writing – review and editing (supporting). **David L. O'Riordan:** data curation (supporting), formal analysis (lead), methodology (supporting), visualization (lead), writing – review and editing (supporting). **Michael W. Rabow:** conceptualization (supporting), funding acquisition (supporting), supervision (supporting), writing – review and editing (supporting). **Adrienne K. Yang:** data curation (supporting), investigation (supporting), writing – review and editing (supporting). **Sohil Patel:** data curation (supporting), investigation (supporting), writing – review and editing (supporting). **Sa Heen Park:** investigation (supporting), writing – review and editing (supporting). **Megan K. McGrath:** investigation (supporting). **Evans M. Whitaker:** conceptualization (supporting), data curation (supporting), methodology (supporting), project administration (supporting), writing – review and editing (supporting). **Sarah S. Nouri:** visualization (supporting), writing – review and editing (supporting). **Stephanie W. Cheng:** conceptualization (equal), data curation (equal), formal analysis (equal), funding acquisition (lead), investigation (equal), methodology (equal), project administration (equal), supervision (lead), visualization (supporting), writing – original draft (supporting), writing – review and editing (lead).

## Conflicts of Interest

The authors declare no conflicts of interest.

## Supporting information


Appendix S1.

## Data Availability

The data that supports the findings of this study are available in Supporting Information of this article.
